# The one-year trajectories of patient-reported outcomes are better for medial unicompartmental knee arthroplasty compared with total knee arthroplasty: a matched cohort study

**DOI:** 10.1302/2633-1462.612.BJO-2025-0164.R1

**Published:** 2025-12-12

**Authors:** Anne Louise Elkjær Christensen, Christian Bredgaard Jensen, Cecilie Henkel, Lina H. Ingelsrud, Kristine I. Bunyoz, Kirill Gromov, Andrew J. Price, Anders Troelsen

**Affiliations:** 1 Clinical Orthopaedic Research Hvidovre, Department of Orthopaedic Surgery, Copenhagen University Hospital, Hvidovre, Denmark; 2 Nuffield Department of Orthopaedics, Rheumatology and Musculoskeletal Science, Nuffield Orthopaedic Centre, Oxford, UK

**Keywords:** Knee, Osteoarthritis, Antero-medial osteoarthritis, Arthroplasty, Knee replacement, Medial unicompartmental knee arthroplasty, Total knee arthroplasty, Patient reported outcome measures, medial unicompartmental knee arthroplasty, patient-reported outcome measures (PROMs), total knee arthroplasty (TKA), Forgotten Joint Score (FJS), Oxford Knee Score, BMI, propensity score matching, preoperative radiographs, linear regression, anteromedial osteoarthritis

## Abstract

**Aims:**

Whether medial unicompartmental knee arthroplasty (mUKA) or total knee arthroplasty (TKA) are more suitable for patients with anteromedial osteoarthritis (AMOA) remains debated. We aimed to compare the development over time in patient-reported outcome measures (PROMs) between patients receiving mUKA and TKA.

**Methods:**

We conducted a matched cohort study of patients receiving TKA or mUKA between March 2018 and February 2020. TKA patients were evaluated based on preoperative radiographs and excluded if not deemed eligible for mUKA. The PROMs (Oxford Knee Score (OKS), Forgotten Joint Score (FJS), and Activity and Participation Questionnaire (APQ)) were completed preoperatively, and at three, six, and 12 months postoperatively. Patients were propensity score matched in a variable 1:2 ratio using BMI, sex, age, and preoperative PROM scores. Area under the curve (AUC) was calculated using the trapezium rule to quantify the change from preoperative PROM scores to scores at three, six, and 12 months postoperatively. AUC differences were analyzed using linear regression.

**Results:**

A total of 618 patients (242 mUKA, 376 TKA) were included. The AUC was significantly lower for TKA patients compared with mUKA patients across all three PROM scores (OKS: ∆AUC of -19% (95% CI -27% to -9 %); FJS: ∆AUC of -23% (95% CI -32% to -14%); APQ -22% (95% CI -32% to -12%). Median PROM scores at three months were 35 and 30 for OKS, 50 and 43 for FJS, 44 and 31 for APQ, for the mUKA and TKA groups, respectively. At six months, the median PROM scores increased to 39 and 35 for OKS, 62 and 50 for FJS, and 56 and 41 for APQ for the mUKA and TKA groups, respectively.

**Conclusion:**

The AUC for PROM scores during the first year was 19% to 23% lower for TKA compared with mUKA. This contrast in development trajectories is present especially in the early recovery three to six months after surgery.

Cite this article: *Bone Jt Open* 2025;6(12):1588–1597.

## Introduction

Total knee arthroplasty (TKA) and medial unicompartmental knee arthroplasty (mUKA) are established surgical treatments for end-stage anteromedial osteoarthritis (AMOA) of the knee.^1,2^ Historically, factors like age, weight, and activity level have limited the use of mUKA. However, some studies have proposed evidence-based and validated criteria without these factors as contraindications.^3,4^ These studies indicate that up to 50% of knee arthroplasty patients may be eligible for mUKA.

mUKA has been associated with higher revision rates compared with TKA.^1^ This may, in part, reflect a lower threshold for revision compared with TKA.^1,5-7^ Revision rates for mUKA have declined over the last 20 years,^8^ and registry data suggest that mUKAs performed by surgeons with high usage (> 20%) have substantially improved implant survivorship.^9-11^ Some studies suggest mUKA offers advantages over TKA for eligible patients. mUKA patients have been shown to have shorter lengths of stay, to be more cost-efficient,^12-16^ and have half the risk of periprosthetic joint infections compared with TKA.^14,17^

Patient-reported outcome measures (PROMs) can provide additional support for treatment choice in value-based healthcare.^1,18^ While existing research, utilizing PROMs, has mainly concentrated on long-term functional outcomes (one to five years), predominantly finding comparable^18-22^ or clinically insignificant differences,^18,23^ some studies have found indications of a potential for better or faster early postoperative recovery for mUKA patients compared with TKA patients.^15,24,25^ These potential differences in early recovery could have meaningful clinical implications, for both patients and healthcare systems. From a patient’s perspective, improved early recovery might influence treatment preferences, despite similar long-term outcomes. Therefore, understanding the potential differences in early recovery following mUKA and TKA could be important to inform treatment choices through shared-decision-making.

In light of this, the objective was to compare the trajectories of development in PROM scores one year postoperatively between mUKA and TKA patients.

## Methods

All patients undergoing primary TKA or mUKA between 12 March 2018 and 27 February 2020 were included. Previous surgery to the same knee was not an exclusion criterion; however, TKA patients with previous anterior cruciate ligament (ACL) reconstruction were excluded to increase comparability, as one of the criteria for mUKA is functionally intact ligaments.^3^ Surgeries were performed at a single centre, university hospital setup (Copenhagen University Hospital Hvidovre, Denmark). Patients with no available PROM data or simultaneous bilateral surgeries were excluded. TKA patients with missing preoperative radiographs were excluded. For patients with two surgeries in the inclusion period, only the second was included. TKA patients deemed ineligible for mUKA based on preoperative radiographs, were excluded to increase group comparability. Radiographs were evaluated twice by the corresponding author (ALEC), using the radiological decision aid by Hamilton et al.^3^ In cases of uncertainty, a consensus decision was reached by the co-authors. Overall, 57.8% of the primary TKAs were deemed radiologically eligible for mUKA. Patient characteristics (sex, age, height, weight) and the American Society of Anesthesiologists (ASA) Physical Status Classification Score^26^ were registered at preoperative physical examination. BMI was calculated from height and weight.

### Surgical technique

Unicompartmental cases were performed using the uncemented medial Oxford partial knee (Zimmer Biomet, USA), with microplasty instrumentation. Indications for mUKA were bone-on-bone AMOA, functionally intact ligaments, a preserved lateral compartment, and no severe lateral facet patellofemoral osteoarthritis. TKA procedures involved implantation of the Nexgen CR, Persona CR, or MC knee systems (Zimmer Biomet), using standard instruments and a measured resection technique with ligament releases as necessary. TKAs were cemented on both tibia and femur. No computer navigation or robotic assistance was used. All TKAs were inserted with the aim to achieve neutral mechanical alignment (proximal tibia cut perpendicular to the long axis and a 5° to 6° valgus cut of the distal femur relative to the long axis). Patellar resurfacing was routinely performed. Surgeries were performed by 11 surgeons, all orthopaedic specialists with subspecialty training in knee arthroplasty: five surgeons performed both mUKA and TKA; while six performed only TKA.

Both groups followed the same standardized postoperative follow-up and rehabilitation protocol, including early mobilization with full weightbearing and access to the same public outpatient rehabilitation services.

### Outcomes

Patients completed the following PROMs: Oxford Knee Score (OKS),^27,28^ Forgotten Joint Score (FJS),^29^ and the OKS Activity and Participation Questionnaire (APQ).^27^ These have demonstrated acceptable levels of validity, reliability, and responsiveness.^27,29-32^ Questionnaires were completed after preoperative physical examination, as well as at three, six, and 12 months postoperatively using Procordo Software (Procordo, Denmark).^33^ Follow-up questionnaires were sent twice by email. If there was no reply or an incomplete reply, a letter containing the questionnaire was sent. Patients without email received the questionnaires by letter once.

### Multiple imputation

Missing values were assumed to be missing at random and handled with the multiple imputation method prior to propensity score matching, using predictive mean matching. Imputation was performed using the mice package in R, creating five imputed datasets.^34^ Missing values for BMI and PROM scores were imputed using surgery type, sex, age, BMI, preoperative PROM score, and three, six, and 12-month PROM scores. A random seed was used for the imputation, and the results presented are based on this original random seed. The imputation was tested using ten different random seeds to ensure consistency. Patients who underwent revision surgery within one year were included in the analysis with imputed data for missing PROM scores to ensure that the results accurately reflected the full range of outcomes.

Missingness before imputation and additional baseline characteristics for responder subgroups, and revised patients, are available in the Supplementary Material.

### Propensity score matching

Patients were propensity score matched in a variable ratio 1:2 (mUKA:TKA) using BMI, sex, age, and preoperative PROM scores (Table I). Propensity scores were estimated using logistic regression. Nearest-neighbour matching without arthroplasty was performed using the MatchIt package in R, with a caliper of 0.2 on the logit scale and TKA as the reference group (MatchIt v. 4.5.3, CRAN; R Foundation for Statistical Computing, Austria). Standardized mean differences (SMDs) of ≤ 0.1 were considered sufficient balance between the groups.^35^

**Table I.** Baseline characteristics.

Variable	Unmatched			Variable 1:2 matched		
	mUKA (n = 244)	TKA (n = 382)	SMD	mUKA (n = 242)	TKA (n = 376)	SMD
Male sex, n (%)	103 (42.2)	165 (43.2)	0.020	103 (42.6)	160 (42.6)	< 0.001
Mean age, yrs (SD)	67.5 (9.3)	67.6 (9.8)	0.015	67.6 (9.3)	67.7 (9.8)	0.013
Median BMI, kg/m^2^ (IQR)	28.6 (25.3 to 32.8)	30.1 (27.6 to 33.2)	0.179	28.7 (25.4 to 33.0)	30.0 (27.6 to 33.1)	0.157
**ASA grade, n (%)**			0.110			0.123
1	15 (6.1)	33 (8.6)		14 (6.8)	33 (8.8)	
2	187 (76.6)	277(72.5)		186 (76.9)	274 (72.9)	
3	42 (17.2)	72 (18.8)		42 (17.4)	69 (18.4)	
Mean OKS score (SD)	23.2 (7.3)	22.46 (6.9)	0.105	23.1 (7.3)	22.5 (6.8)	0.090
Median FJS score (IQR)	14.6 (6.08 to 25)	13.6 (4.17 to 25)	0.053	14.6 (6.3 to 25.0)	13.6 (4.2 to 25.0)	0.050
Median APQ score (IQR)	6.3 (3.1 to 18.8)	9.4 (3.1 to 18.8)	0.048	6.3 (3.1 to 18.8)	9.38 (3.1 to 18.8)	0.022

Baseline patient characteristics, unmatched and propensity score matched in a variable ratio 1:2 using BMI, sex, age, and preoperative patient-reported outcome measure scores.

FJS 0 to 100, OKS 0 to 48, APQ 0 to 100.

APQ, activity and participation questionnaire; ASA, American Society of Anesthesiologists; FJS, Forgotten Joint Score; mUKA, medial unicompartmental knee arthroplasty; OKS, Oxford Knee Score; SMD, standardized mean difference; TKA, total knee arthroplasty.

### AUC

Changes in PROM scores from baseline were calculated using the trapezium rule, as a measure of improvement over time.^36,37^ AUC, with time on the x-axis and PROM scores on the y-axis, represents the average change in PROMs over the follow-up period.^38^ The between-group differences in AUC were compared using linear regression, unadjusted and adjusted for preoperative scores, ASA, sex, and age. Estimates for the difference in AUC were expressed as a percentage, illustrating the proportional difference in AUC for TKA patients compared with mUKA patients.

### Probability of negative PROM change

The proportion of patients with a negative treatment effect on PROM scores was calculated using Fisher’s exact test, stratified by surgery type. Negative treatment effect was defined as lower postoperative PROM scores compared with preoperative scores, or a negative AUC. The odds ratio for a TKA patient experiencing a negative change in PROM score was calculated relative to mUKA.

### Statistical analysis

Baseline characteristics were assessed using histograms and quantile-quantile plots. Categorical data are displayed as n (%). Continuous data are summarized as mean (SD) or median (IQR). Statistical significance was defined as p-values < 0.05. Analyses were performed using R v. 4.1.0 (R Foundation for Statistical Computing).

## Results

**Fig. 1:**
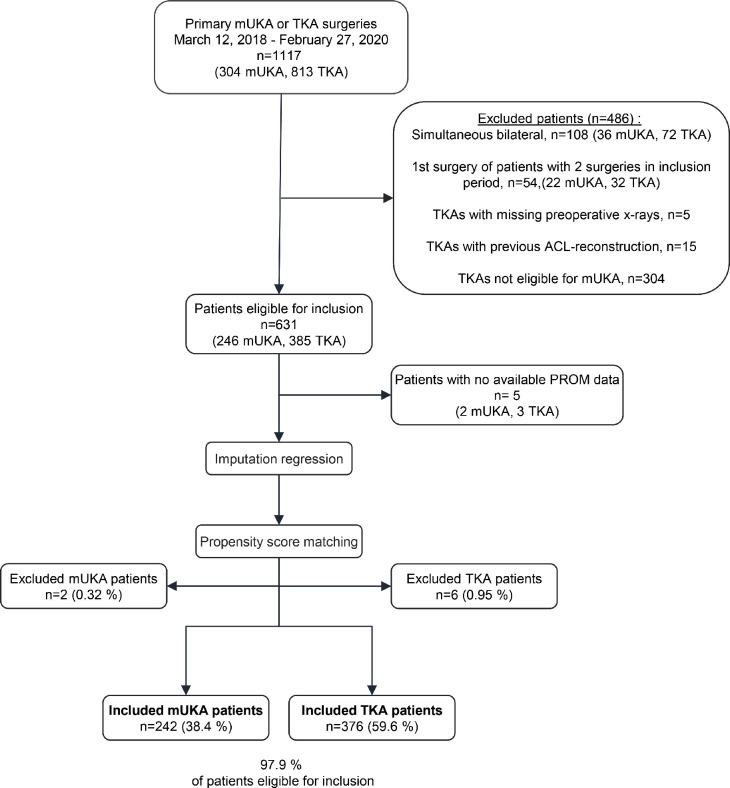
Flowchart of final study population. Included patients underwent primary total knee arthroplasty (TKA) or medial unicompartmental knee arthroplasty (mUKA) at Copenhagen University Hospital Hvidovre between 12 March 2018 and 27 February 2020. The exclusion criteria was simultaneous bilateral surgery, missing patient-reported outcome measure (PROM) data at all time points, TKA patients with previous anterior cruciate ligament (ACL) reconstruction, or with missing preoperative radiographs and TKA patients not eligible for mUKA. Additionally, the first surgery of patients with two surgeries in the inclusion period were excluded.

Data from 1,117 patients (304 mUKA, 813 TKA) were available. Figure 1 shows the inclusion and exclusion process. Ultimately, 618 patients (242 mUKA, 376 TKAs) were included in the analysis.

Prior to imputation, 274 patients had missing values for either BMI or PROM scores. Thus, 357 of the 631 patients had complete data, rendering a response rate of 56.6%. Proportion of missingness prior to imputation is shown in Supplementary Material.

Baseline characteristics, before and after matching, are shown in Table I. After matching, the groups had sufficient balance on all matched parameters, except BMI, where TKA patients had a higher median BMI, 30.0 kg/m^2^ (IQR 27.6 to 33.1), compared with mUKA patients, 28.7 kg/m^2^ (25.4 to 33.0).

### AUC

**Fig. 2:**
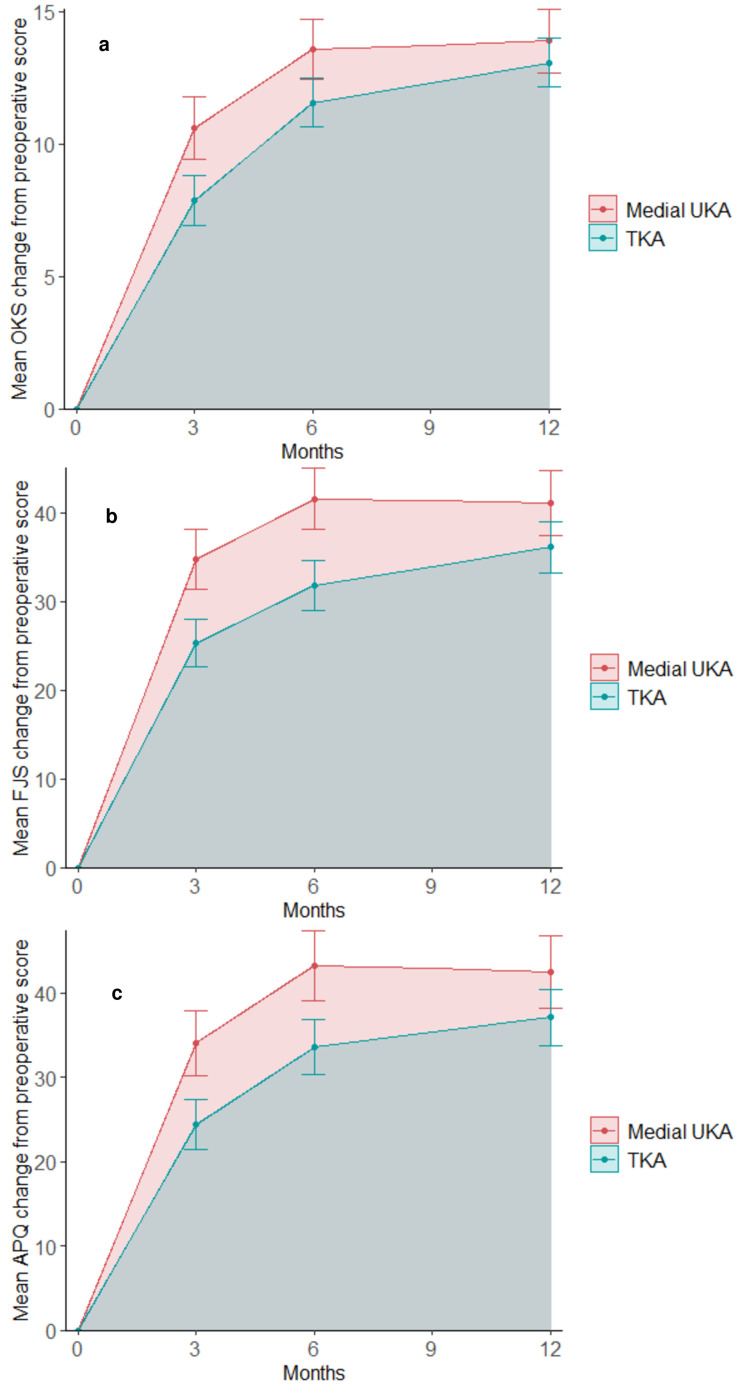
One-year trajectory of patient-reported outcome measure (PROM) scores (Oxford Knee Score (OKS), Forgotten Joint Score (FJS), activity and participation questionnaire (APQ)). This figure illustrates the mean change from preoperative PROM score, stratified for medial unicompartmental knee arthroplasty (mUKA) and total knee arthroplasty (TKA). The x-axis displays months after surgery, and the y-axis represents mean change from preoperative scores. Error bars denoting the 95% CI of the mean. a) OKS; b) FJS c) APQ.

The AUC was significantly lower for TKA patients compared with mUKA patients across all three PROMs, unadjusted and adjusted for preoperative PROM scores, ASA score, sex, and age (Table II, Figure 2). Mean AUC, calculated from individual AUCs for each patient, showed a greater area of development for mUKA patients in all three PROMs. Adjusted proportional differences in AUC, were -19% lower for TKA compared with mUKA in OKS (95% CI -27% to -9%); -23% lower for TKA in FJS (95% CI -32% to -14%), and -22% lower for TKA in APQ (95% CI -32% to -12%).

**Table II.** Area under the curve regression results.

Score	Mean mUKA, AUC (SD)	Mean TKA, AUC (SD)	∆AUC estimate (crude)	95% CI	p-value	∆AUC estimate (adjusted)*	95% CI	p-value
**OKS**	130 (82.9)	111 (84.3)	-19	-32 to -5	< 0.01	-24	-35 to -12	< 0.001
Percentage difference			-14.0	-25% to -4%		-19.0	-27 to -9	
**mUKA**	400 (258)	317 (255)	-83	-125 to -42	< 0.001	-92	-129 to -55	< 0.001
Percentage difference			-21.0	-31 to -11		-23.0	-32 to -14	
**APQ**	408 (296)	325 (288)	-83	-130 to -36	< 0.001	-89	-131 to -47	< 0.001
Percentage difference			-20.0	-32 to -9		-22.0	-32 to -12	

Areas under the curve (AUCs) were calculated to measure improvement in patient-reported outcome measures (PROMs) over time. Estimates for AUC differences were compared using linear regression, both crude and adjusted for preoperative scores, sex, and age. Percentages illustrate the proportional difference in AUC for total knee arthroplasty patients compared with medial unicompartmental knee arthroplasty patients.

* Adjusted for preoperative score, sex, age, and American Society of Anesthesiologists score.

APQ, activity and participation questionnaire; AUC, area under the curve; FJS, Forgotten Joint Score; mUKA, medial unicompartmental knee arthroplasty; OKS, Oxford Knee Score; TKA, total knee arthroplasty.

A total of 15 patients (seven mUKA, eight TKA) underwent revision surgery within one year. Additional AUC analyses, excluding revised patients, and those with imputed data were consistent with the primary findings.

### Median PROM scores

**Fig. 3:**
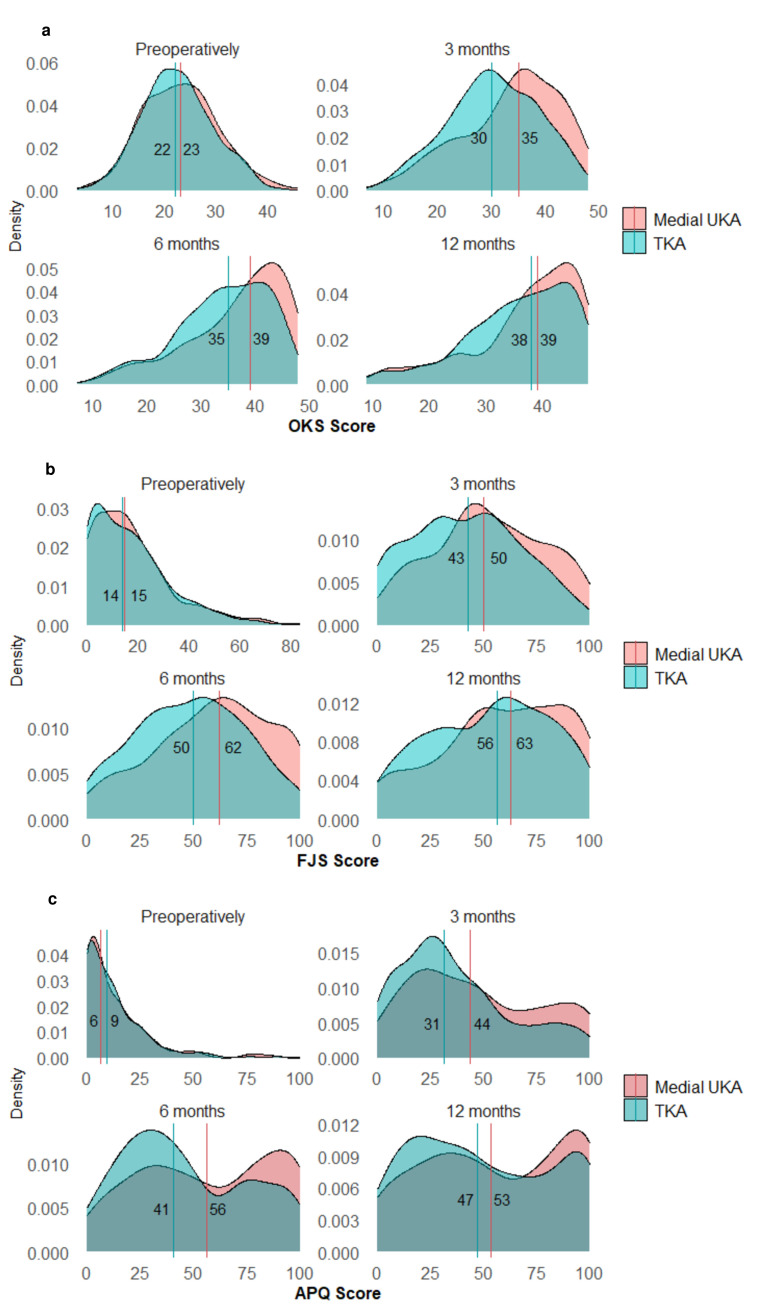
Multivariate kernel density plot of patient-reported outcome measure (PROM) scores (Oxford Knee Score (OKS), Forgotten Joint Score (FJS), activity and participation questionnaire (APQ)). Density plots, showing the density of responses in PROM scores preoperatively, three to six and 12 months postoperatively, stratified for medial unicompartmental knee arthroplasty (mUKA) and total knee arthroplasty (TKA). Vertical lines represent median scores, for mUKA (red) and TKA (blue). a) OKS; b) FJS; c) APQ.

The median PROM scores at three months were 35 and 30 for OKS, 50 and 43 for FJS, 44 and 31 for APQ, for the mUKA and TKA groups, respectively. At six months, the median PROM scores increased to 39 and 35 for OKS, 62 and 50 for FJS, and 56 and 41 for APQ, for the mUKA and TKA groups, respectively. At 12 months median PROM scores increased to 39 and 38 for OKS, 63 and 56 for FJS, and 53 and 47 for APQ, for the mUKA and TKA groups, respectively (Figure 3). The most notable difference between mUKA and TKA was seen three to six months postoperatively, favouring mUKA.

### Probability of negative PROM change

The proportion of patients with a negative treatment effect on PROM scores was higher for TKA than mUKA in both AUC and at three, six, and 12 months after surgery for all three PROM scores, except APQ score at 12 months (Table III). However, at 12 months, the odds ratios for a TKA patient experiencing a negative treatment effect relative to a mUKA patient were close to 1 across all PROM scores, indicating minimal or no differences between groups at this time point.

**Table III.** Probability of negative treatment effect.

Probability of a negative treatment effect on OKS												
	**∆AUC**			**3 months**			**6 months**			**12 months**		
	**%**	**OR**	**95% CI**	**%**	**OR**	**95% CI**	**%**	**OR**	**95% CI**	**%**	**OR**	**95% CI**
UKA	6.2	Ref		12.4	Ref		6.61	Ref		7.44	Ref	
TKA	8.78	1.46	0.75 to 2.95	18.4	1.59	0.98 to 2.62	11.2	1.8	0.98 to 3.44	8.24	1.12	0.59 to 2.18
**Probability of a negative treatment effect on FJS**												
	**∆AUC**			**3 months**			**6 months**			**12 months**		
	**%**	**OR**	**95% CI**	**%**	**OR**	**95% CI**	**%**	**OR**	**95% CI**	**%**	**OR**	**95% CI**
UKA	5.37	Ref		8.26	Ref		7.02	Ref		8.68	Ref	
TKA	8.78	1.69	0.85 to 3.59	16.2	2.15	1.24 to 3.87	12	1.60	0.85 to 3.09	9.57	1.11	0.61 to 2.06
**Probability of a negative treatment effect on APQ**												
	**∆AUC**			**3 months**			**6 months**			**12 months**		
	**%**	**OR**	**95% CI**	**%**	**OR**	**95% CI**	**%**	**OR**	**95% CI**	**%**	**OR**	**95% CI**
UKA	7.44	Ref		9.5	Ref		6.2	Ref		9.5	Ref	
TKA	9.31	1.28	0.68 to 2.46	12.8	1.39	0.80 to 2.47	11.7	2.0	1.06 to 3.97	8.78	0.92	0.51 to 1.68

Negative treatment effect defined as patients having lower postoperative patient-reported outcome measure (PROM) scores compared with preoperative PROM scores. Odds ratio (OR) was calculated as the odds of a total knee arthroplasty patient experiencing a negative treatment effect relative to a medial unicompartmental knee arthroplasty patient.

AUC, area under the curve; FJS, Forgotten Joint Score; OKS, Oxford Knee Score.

The biggest difference was seen at three and six months postoperatively. In OKS, 30 mUKA patients (12.4%) had a negative treatment effect at three months compared with 69 TKA patients (18.4%), and at six months, it was 16 (6.6%) for mUKA compared with 42 (11.2%) for TKA. In FJS, the proportion of patients with a negative treatment effect was 8.3% (n = 20) for mUKA compared with 16.2% (n = 61) for TKA at three months, and at six months the results were 7.0% (n = 17) for mUKA compared with 12.0% (n=45) for TKA. In APQ, 23 mUKA patients (9.5%) had a negative treatment effect at three months compared with 48 (12.8%) for TKA, and 15 (6.2%) for mUKA compared with 44 (11.7%) for TKA at six months. In contrast, the proportion of mUKA patients with a negative treatment effect on APQ at 12 months was slightly higher compared with TKA, with 23 (9.5%) for mUKA, and 33 (8.8%) for TKA. Statistically significant differences were, however, only found in FJS at three months and APQ at six months, both favouring mUKA.

### Distribution of high scorers

A higher proportion of mUKA patients reached the highest possible scores compared to TKA on all three PROM scores. In OKS, 15 (6.1%) mUKA and 15 TKA patients (4%) reached the highest possible scores. In FJS, it was 11.6% (n = 28) for mUKA, and 6.4% (n = 24), for TKA patients. In APQ distribution, it was 18.6% (n = 45) of the mUKA, and 10.4% ( n= 39) of the TKA patients.

## Discussion

This study showed mUKA patients had significantly better early functional recovery within 12 months compared with TKA, with TKA patients demonstrating 18% to 23% lower improvement in PROM scores, measured in AUC of OKS, FJS, and APQ. Additionally, mUKA patients had higher median PROM scores, especially at three and six months.

To our knowledge, this is the first comparison of the early one-year postoperative trajectory of mUKA and TKA surgery using difference in AUC, making it difficult to compare directly with other studies. The use of AUC may provide a more nuanced insight into the trajectory of recovery, compared with traditional single-point assessments, as it reflects a time-weighted average of PROM scores across follow-up time points. The TOPKAT study used AUC of mean OKS as a secondary outcome, showing a 1.54 points lower AUC of mean OKS score for TKA patients compared with mUKA patients, but this was calculated based on all data available from the five-year follow-up period.^15^ As most of the functional recovery appears to occur within the first year, a 12-month AUC approach may offer a more focused and clinically relevant insight into differences in early postoperative outcomes.

Both groups showed improvements in OKS from baseline that exceeded the minimal important change (MIC) thresholds for UKA and TKA at both three and 12 months.^39^ This indicates that both procedures led to patient-perceived meaningful change. Currently, there is no established minimal clinically important difference in AUC for OKS, FJS, or APQ. Still, our results are supported by findings of the TOPKAT study, which indicate a stagnation in functional recovery for both mUKA and TKA within the first year.^15^ Additionally, a 2021 randomized controlled trial showed faster functional recovery for mUKA patients, with a 6.2 mean between-group difference in OKS score two months after surgery, although no difference were found at two years.^24^ The findings of the present study, however, contrast with those of Sershon et al,^20^ who found no significant difference six weeks and six months after surgery in FJS, Knee Injury score, and Osteoarthritis Outcome Score (KOOS), and Knee Society Score (KSS).

While existing research suggests comparable long-term functional outcomes for mUKA and TKA,^19-22^ the present study focuses instead on short-term functional recovery. The results, indicating a faster recovery for mUKA patients, may add nuance to discussions of surgical treatment options. While the clinical implications of early PROM improvements remain to be fully understood, they could be relevant to certain patient groups prioritizing early postoperative function.

Being a retrospective cohort study, the absence of randomization may introduce imbalance in baseline characteristics. Selection-bias could be considered a potential limitation, as some argue mUKA is more likely to be chosen for healthier patients.^6^ After matching on BMI, sex, age, and preoperative PROM scores, the TKA group still had a 1.3 higher median BMI and a slightly higher proportion of ASA 1 patients. The AUC calculations were adjusted for preoperative PROM scores, ASA score, sex, and age, to minimize the impact of remaining imbalances. However, residual confounding from unknown variables cannot be definitively ruled out.^40^

Another limitation of this study is that all mUKAs performed during the inclusion period were uncemented Oxford implants, which may limit generalizability of the findings to cemented or other implant designs.

Variation in surgeon groups, with five performing both mUKA and TKA, and six performing only TKA, presents a potential source of bias. Matching was used to minimize this, but differences in surgeon experience and learning curves may still have influenced outcomes.

When using PROMs, missing response data is a limitation. Five patients were excluded due to missing data at all time points. The proportion with complete data was 56.6% and may cause some selection-bias. This was addressed by using multiple imputation. Comparing mean PROM scores before and after imputation showed a slight decrease in postoperative scores for both groups, suggesting a potential bias where patients with poorer outcomes are less inclined to respond to postoperative questionnaires. A 2022 study on THA, using the FJS questionnaire, identified a similar tendency.^41^ By using multiple imputation, we tried to mitigate this bias, although some degree of non-responder bias cannot be fully excluded due to the moderate response rate.

A higher proportion of mUKA patients reached the highest possible scores, on all three PROM scores. Specifically, 18.6% of mUKA patients reached the highest possible score in APQ, exceeding the 15% threshold commonly defined for problematic ceiling effect.^32,42,43^ Ceiling effect reduces the ability to accurately measure improvement, for those scoring at the highest levels.^44^ This study showed mUKA patients had the highest scores and improvement in APQ compared with TKA patients. Thus, the observed difference in APQ, favouring mUKA, may potentially be higher than what APQ can measure, as the highest scorers may be constrained by ceiling effect.

The findings of this study indicate that functional recovery predominantly happens within three to six months after surgery. Future research could therefore be recommended to focus on short-term recovery, alongside revision rates for mUKA and TKA, as there is a possibility for early differences that might be of importance to patients.

In conclusion, Oxford mUKA patients had significantly better early functional recovery within the first 12 months compared with TKA patients. Although previous studies have demonstrated comparable long-term outcomes, these findings of differences in early functional recovery may be relevant for surgeons to consider, when discussing surgical treatment options with patients.

**Take home message**

- Medial unicompartmental knee arthroplasty (mUKA) patients demonstrate significantly better early postoperative functional recovery compared to total knee arthroplasty (TKA) patients.

- The majority of functional recovery for both mUKA and TKA patients occurs within the first three to six months postoperatively, suggesting that short-term recovery may be a key differentiating factor between the two procedures.

## Data Availability

The data that support the findings for this study are available to other researchers from the corresponding author upon reasonable request.
